# Correlation Between Intravascular Ultrasound Minimum Luminal Area and Quantitative Minimum Luminal Area in Intermediate Coronary Lesions

**DOI:** 10.7759/cureus.81924

**Published:** 2025-04-08

**Authors:** Mohammad Mohammadi, Hamed Bazrafshan Drissi, Mahdi Rahmanian, Javad Kojuri, Armin Attar, Ali Safari, Mehdi Bazrafshan, Peyman Izadpanah

**Affiliations:** 1 Atherosclerosis Research Center, Ahvaz Jundishapur University of Medical Sciences, Ahvaz, IRN; 2 Cardiovascular Research Center, Shiraz University of Medical Sciences, Shiraz, IRN; 3 Department of Cardiology, School of Medicine, Shiraz University of Medical Sciences, Shiraz, IRN

**Keywords:** acute coronary syndrome, coronary artery angiography, interventional ultrasonography, myocardial infarction, primary percutaneous coronary intervention (pci)

## Abstract

Background: Chronic coronary syndrome (CCS) is a common disease worldwide. Advances in technology, including quantitative coronary angiography, have improved CCS management. In the present study, we aim to investigate the correlation and agreement between intravascular ultrasound minimum luminal area (IVUSMLA) and quantitative minimum luminal area (QMLA) in CCS patients with intermediate coronary lesions.

Methods: Data from CCS patients with at least one-vessel coronary occlusion from March 2022 to March 2023 were collected. Gathered data included patients' age, gender, QMLA, IVUSMLA, and patients' previous medical history, including hypertension, diabetes, and smoking. The target lesion in this study was intermediate (50% to 70%) stenosis. Agreement between IVUSMLA and QMLA was assessed using the Bland-Altman plot. A p-value (p) of less than 0.05 was considered statistically significant. R version 4.4.1 software (R Foundation for Statistical Computing, Vienna, Austria) was used for data analysis.

Results: This study included 52 (59.1%) men and 36 (40.9%) women with a mean age of 67.15 ± 10.63 and 64 ± 12.61 (p = 0.533), respectively. The mean for IVUSMLA and QMLA was 2.80 ± 1.54 and 2.6 ± 1.57, respectively. QMLA was highly correlated with IVUSMLA (r = 0.997; p <0.001). Both QMLA and IVUSMLA had moderately good agreement, presenting with small mean difference values but large values of root mean squared deviation (IVUSMLA - QMLA = 0.13 ± 0.11).

Conclusion: IVUSMLA and QMLA are highly correlated to each other and have good agreement. So, QMLA can be used instead of IVUSMLA in intermediate coronary lesions.

## Introduction

Chronic coronary syndrome (CCS) is a common disease worldwide [1]. Equipment and techniques have improved the management of CCS and patients' clinical outcomes over the past few decades [2].

Invasive coronary angiography plays a vital role in CCS management. It confirms lesions in coronary arteries and allows for treatment using medical therapy alone or medical therapy plus percutaneous coronary intervention (PCI) [3].

Based on angiographic results, the percentage of coronary artery blockage can be calculated and categorized into mild, intermediate, or severe. It has been challenging for cardiologists to decide whether or not angioplasty is required in cases with intermediate blockage. Fractional flow reserve (FFR) is a guide-wire-based technique that makes it possible to measure blood pressure and flow through a segment of the coronary artery. Regarding intermediate blockage, if FFR reveals good blood flow (FFR equal to or greater than 0.8), revascularization will not be necessary [4,5].

Intravascular ultrasound (IVUS) is an imaging modality with both clinical and research applications, used to visualize the coronary artery wall and characterize plaque morphology and distribution, quantify plaque burden, guide stent sizing, assess stent expansion, and reduce procedural complications [6]. PCI under the IVUS guide is recommended to be safer and more promising. IVUS-guided PCI has been associated with improved stent expansion, reduced restenosis, and better long-term outcomes. Also, IVUS can provide clarity where angiography results are vague or ambiguous, including intermediate lesions of uncertain stenotic severity [7-9]. IVUS minimum luminal area (IVUSMLA) is one of many determinants of coronary artery blockage severity that has been proposed as an alternative to FFR to assess coronary artery disease (CAD) severity, including evaluating intermediate lesions [10,11].

Although FFR and IVUS procedures have made decision-making over intermediate blockage easy, they are invasive and possibly impose more complications on patients. Therefore, a noninvasive option to calculate the intermediate blockage flow rate seems interesting. In a clinical setting, quantitative coronary angiography (QCA) can be used to evaluate coronary artery stenosis. QCA is based on conventional contrast angiography and specific software that helps with the determination of some specific measures of coronary arteries, e.g., quantitative flow ratio (QFR) and quantitative minimum luminal area (QMLA), in an operator-independent way [12]. QFR is a novel technique to assess the pressure drop in coronary arteries based on two angiographic projections. It has been proven a safe and cost-effective alternative to FFR [13,14]. Moreover, QFR has been associated with QMLA [15].

The Iranian population is composed of diversified ethnic groups with a high prevalence of CADs, including many patients suffering from intermediate coronary artery lesions [16]. So, in this study, we aim to investigate the correlation and agreement of QMLA and IVUSMLA in patients with at least one-vessel CAD in an Iranian sample in a cardiology referral center in south Iran to find out if we can replace IVUSMLA with QMLA.

## Materials and methods

Study design

Patients with CCS symptoms were studied between March 2022 and March 2023, referring to outpatient clinics of Faghihi and Al-Zahra hospitals affiliated with Shiraz University of Medical Sciences, Shiraz, Iran. Patients are selected based on their prior visits and diagnoses at outpatient clinics. Based on the results of noninvasive testing for CAD, including nuclear imaging, exercise stress test, and coronary CT angiography in outpatient clinics, patients likely to have CCS were selected and became candidates for angiography. Right after the angiography, IVUS and QCA were performed in patients suspected of having intermediate coronary lesions. The target lesion in this study was intermediate (50% to 70%) stenosis, which was visually estimated using angiography. We included patients with at least a one-vessel lesion. Also, those patients with previous coronary interventions, including angioplasty or bypass graft, patients with ST-segment elevation myocardial infarction (STEMI), and non-STEMI patients, as well as patients with chronic total occlusion, were excluded.

Patients' data including gender, age, type of occluded vessel, PCI result, IVUSMLA, quantitative reference area (QRFA), QMLA, quantitative area stenosis (QAS), quantitative diameter stenosis (QDS), and prior medical history of patients including hypertension, diabetes mellitus, and smoking were gathered. This study was approved by the Ethics Committee of Shiraz University of Medical Sciences (IR.SUMS.MED.REC.1401.505). All methods were performed according to the relevant guidelines and regulations. Informed consent was obtained from all subjects and/or their legal guardians.

Coronary angiography and IVUS

Coronary angiography was done with a trans-femoral or trans-radial approach using a 5-Fr or 6-Fr catheter. In order to lower the heart rate to around 60 beats per minute or less, patients with higher heart rates received 50 to 100 mg of oral metoprolol before the procedure. Intra-arterial opacification of approximately 250 HU was injected at 5 to 7 cc per second. Angiograms were obtained using a monoplane radiographic system. Intracoronary nitroglycerin was injected before every angiography. Two angiographic views with at least a 25-degree difference were obtained for all suspected lesions, and the baseline frame rate was at least 15 per second for all procedures. For arteries with 50% to 70% occlusion visually, IVUS assessment with automatic pullback of 2.0 mm/s and frequency of 40 MHz was performed (VOLKANO, Trillium Technology, San Diego, CA, US).

Three-dimensional QCA

A trained, certified specialist (MM) who was blind to the IVUS results of patients performed quantitative coronary angiographic analysis using software (Q Angio XA 3D version 2.1.38.2, Medis Medical Imaging System, Leiden, the Netherlands). Two angiographic projections at least 25 degrees apart were used for analysis. The software reconstructed a three-dimensional anatomic vessel model to calculate QCA determinants, including QFR, QMLA, QAS, QDS, and QRFA. QFR lower than 0.8 is considered significant stenosis.

Statistics

Descriptive data were presented as the mean ± standard deviation (SD), frequency, and percentage. The chi-squared test and independent sample t-test were used for bivariate analysis. Pearson correlation was used to quantify the correlation between IVUSMLA and QMLA. Agreement between IVUSMLA and QMLA was assessed using the Bland-Altman plot. The Bland-Altman plot shows the difference between each pair of values versus their mean value. The limits of agreement were defined as mean ± 1.96 SD of absolute difference. A p-value (p) of less than 0.05 was considered statistically significant. R version 4.4.1 software (R Foundation for Statistical Computing, Vienna, Austria) was used for data analysis.

## Results

In total, 88 patients were studied, including 52 (59.1%) men and 36 (40.9%) women. The mean age of men and women was 67.15 ± 10.63 and 64 ± 12.61, respectively (p = 0.533). Women had more prior history of diabetes than men (p < 0.001), while the number of smokers was more frequent among men than women (p < 0.001). PCI was performed on 24 (46.1%) men and 16 (44.4%) women (p = 0.937) (Table 1).

**Table 1:** Patients’ demographics *Independent-sample t and chi-squared tests were used. p-value < 0.05 is considered statistically significant SD: standard deviation; PCI: percutaneous coronary intervention

Item	Male (N = 52)	Female (N = 36)	p-value^*^
Age (mean ± SD)	67.15 ± 10.63	64 ± 12.61	0.533
Hypertension	32 (62.5%)	20 (55.6%)	0.575
Diabetes mellitus	8 (15.4%)	18 (50%)	<0.001
Smoker	40 (76.9%)	5 (13.9%)	<0.001
PCI	24 (46.1%)	16 (44.4%)	0.937

The most frequent lesions were detected in the left anterior descending artery (LAD) (N = 64, 72.7%). The mean for IVUSMLA and QMLA was 2.80 ± 1.54 and 2.6 ± 1.57, respectively (Table 2).

**Table 2:** Vessel and lesion characteristics LAD: left anterior descending artery; LCX: left circumflex coronary artery; RCA: right circumflex artery; IVUS: intravascular ultrasound; IVUSMLD: intravascular ultrasound minimum luminal area; QRFA: quantitative reference area; QMLA: quantitative minimum luminal area; AS: area stenosis; DS: diameter stenosis; QFR: quantitative flow ratio

Vessel (N (%))	
LAD	64 (72.7%)
LCX	12 (13.6%)
RCA	12 (13.6%)
IVUS index	
IVUSMLD (mean ± SD (mm))	2.80 ± 1.54
Quantitative coronary angiographic indexes	
QRFA (mean ± SD (mm))	5.77 ± 1.80
QMLA (mean ± SD (mm))	2.6 ± 1.57
QFR (mean ± SD)	0.77 ± 0.16
AS (%)	55.27 ± 16.5
DS (%)	44.55 ± 12.65

**Figure 1:**
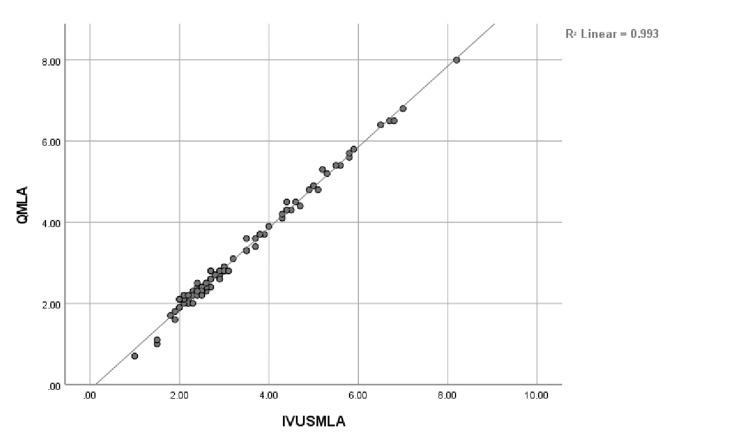
Correlation between quantitative minimum luminal area (QMLA) and intravascular ultrasound minimum luminal area (IVUSMLA) (N = 88)

**Figure 2:**
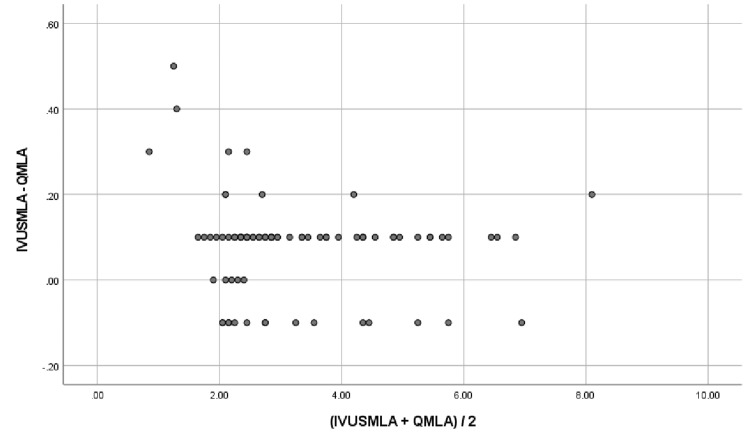
Agreement between quantitative minimum luminal area (QMLA) and intravascular ultrasound minimum luminal area (IVUSMLA) (N = 88)

**Figure 3:**
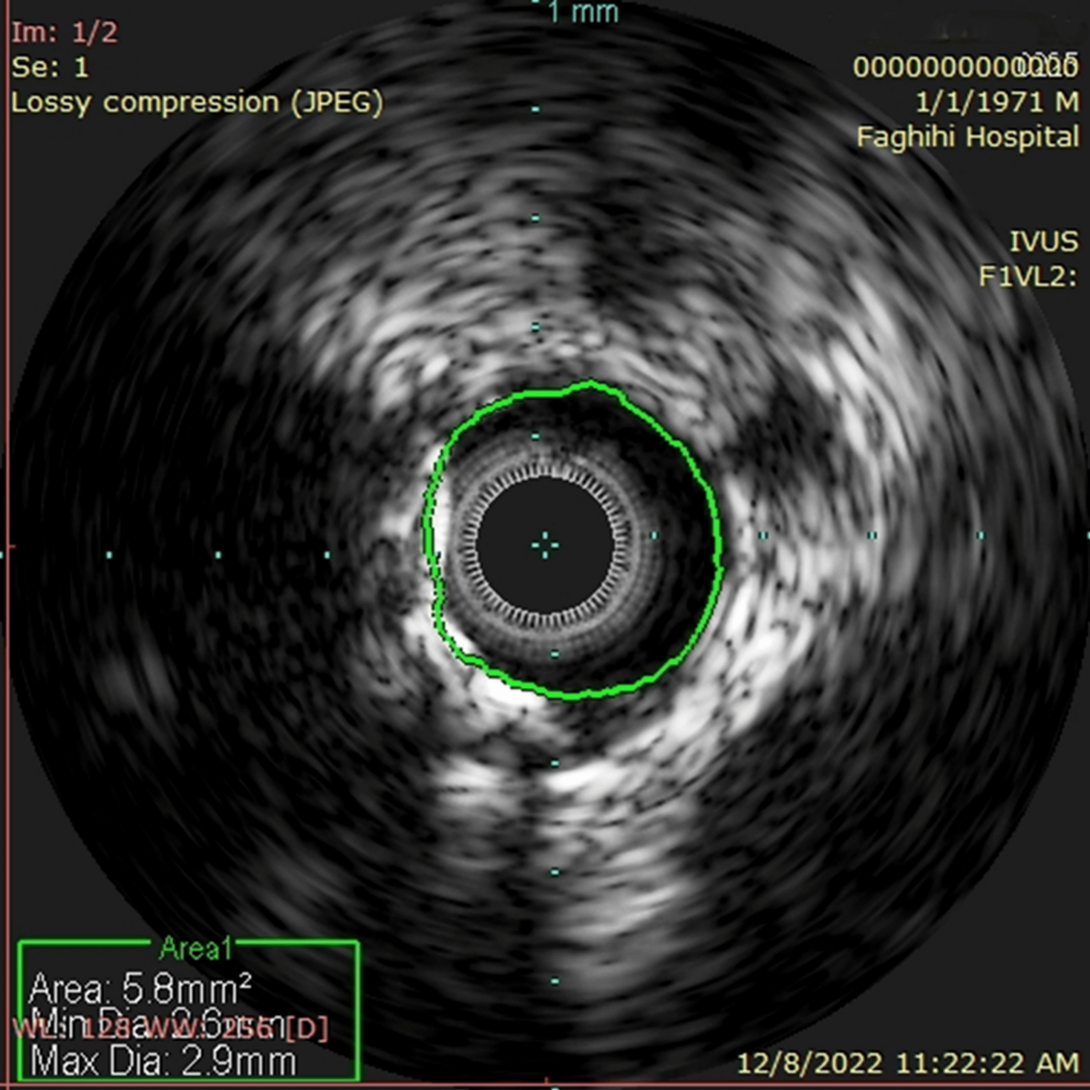
Intravascular ultrasound minimum luminal area (IVUS) assessment picture from one of the studied patients

A representative example of QMLA and IVUSMLA measurements is shown in Figures 1, 2. QMLA was highly correlated with IVUSMLA (r = 0.997; p <0.001). Both QMLA and IVUSMLA had moderately good agreement, presenting with small mean difference values but large values of root mean squared deviation (IVUSMLA - QMLA = 0.13 ± 0.11). Figure 3 shows an IVUS assessment picture from one of the studied patients.

## Discussion

This study found that women and men with CAD were of the same age. Diabetes mellitus was more common among women than men; most smokers were men. The main finding of our study was that IVUSMLA and QMLA were highly correlated and had a good agreement. In concordance with the result of our study, Lee et al. also found that there is no significant difference between IVUSMLA and QMLA measures [17]. As measuring IVUSMLA is an invasive, expensive, and time-consuming process that might be adversely affected by visual artifacts diminishing its accuracy, QMLA seems to be a better noninvasive, cheaper, and more available tool in the assessment of intermediate coronary lesions.

Previous studies have suggested that PCI optimization by IVUS leads to better post-PCI outcomes. In their randomized case-control trial, Neleman et al. reported that IVUS-guided PCI significantly improved post-PCI FFR [18]. In another cohort study, Mentias et al. claimed that IVUS guidance in PCI is associated with lower long-term mortality, myocardial infarction (MI), and repeat revascularization [19]. Some prior studies showed that IVUS results are comparable to FFR results, meaning they can be used interchangeably. Koo et al. revealed in their study that in patients with intermediate stenosis who were being evaluated for PCI, both FFR guidance and IVUS guidance concerning mortality, MI, or revascularization were similarly promising [20]. Yang et al. showed no difference in IVUS characteristics between patients with borderline or severe FFR regarding MLA, lesion length, plaque burden, plaque volume, and amount of atheromatous volume [21]. Regarding the current study's and previous research findings, we believe that performing QCA before and after PCI, especially calculating QMLA in patients suffering from intermediate coronary lesions, might enhance the PCI outcome.

On the one hand, IVUS and FFR techniques have shown promising results in estimating coronary artery blockage. On the other hand, they are both invasive and expensive procedures. In the past few years, researchers have tried to understand whether or not the determinants obtained from QCA are as promising and reliable. The multicentric FAVOR II Europe-Japan Study reported that computation of QFR in the catheterization laboratory is clinically superior to angiographic assessment to evaluate intermediary coronary artery stenosis using FFR [22]. Moreover, the multicenter, randomized, controlled FAVOR III China trial showed that QFR-guided lesion selection improved two-year clinical outcomes compared with standard angiography guidance [23]. Also, Tanigaki et al. showed that QFR has a high correlation and a good agreement with FFR [24]. Pepper et al. also reported a good correlation between QFR and FFR [25].

In this study, we aimed to investigate the correlation and agreement between IVUSMLA and QMLA. As they are strongly correlated, we recommend using QMLA-a noninvasive, cheaper, and accurate modality-to evaluate intermediate coronary lesions.

Limitations

Our study was observational. Also, our study design lacked follow-up after the intervention to evaluate patients for possible complications. Moreover, if the study sample size was larger, we could probably report a better agreement between the two tools. Besides, the study design was single-center and did not include a comparison of IVUSMLA and QMLA in different vessels, subgroups, and genders.

## Conclusions

In conclusion, IVUSMLA and QMLA are highly correlated to each other and have good agreement. So, QMLA can potentially be a precise substitution for IVUSMLA in evaluating intermediate coronary lesions. A future study using a larger cohort of patients with follow-up after the intervention, comparing FFR with QMLA, and including the cost-effectiveness analysis is recommended to be conducted.

## References

[1] Cassar A, Holmes DR Jr, Rihal CS, Gersh BJ (2009). Chronic coronary artery disease: diagnosis and management. Mayo Clin Proc.

[2] Prati F, Di Vito L, Biondi-Zoccai G (2012). Angiography alone versus angiography plus optical coherence tomography to guide decision-making during percutaneous coronary intervention: the Centro per la Lotta contro l'Infarto-Optimisation of Percutaneous Coronary Intervention (CLI-OPCI) study. EuroIntervention.

[3] von Koch S, Koul S, Grimfjärd P (2024). Percutaneous coronary intervention plus medical therapy versus medical therapy alone in chronic coronary syndrome: a propensity score-matched analysis from the Swedish Coronary Angiography and Angioplasty Registry. Heart.

[4] Hill D, Bykowski A, Lim MJ (2025). Fractional flow reserve. StatPearls [Internet].

[5] Mohdnazri SR, Keeble TR, Sharp AS (2016). Fractional flow reserve: does a cut-off value add value?. Interv Cardiol.

[6] Parviz Y, Shlofmitz E, Fall KN (2018). Utility of intracoronary imaging in the cardiac catheterization laboratory: comprehensive evaluation with intravascular ultrasound and optical coherence tomography. Br Med Bull.

[7] di Mario C, Koskinas KC, Räber L (2018). Clinical benefit of IVUS guidance for coronary stenting: the ULTIMATE step toward definitive evidence?. J Am Coll Cardiol.

[8] Saito Y, Kobayashi Y, Fujii K (2022). Clinical expert consensus document on intravascular ultrasound from the Japanese Association of Cardiovascular Intervention and Therapeutics (2021). Cardiovasc Interv Ther.

[9] Viana SM, Zhang DM (2024). Intravascular ultrasound guiding percutaneous coronary interventions in complex higher risk-indicated patients (CHIPs): insight from clinical evidence. Rev Cardiovasc Med.

[10] Nascimento BR, de Sousa MR, Koo BK, Samady H, Bezerra HG, Ribeiro AL, Costa MA (2014). Diagnostic accuracy of intravascular ultrasound-derived minimal lumen area compared with fractional flow reserve--meta-analysis: pooled accuracy of IVUS luminal area versus FFR. Catheter Cardiovasc Interv.

[11] Cooper BZ, Kirwin JD, Panetta TF (2001). Accuracy of intravascular ultrasound for diameter measurement of phantom arteries. J Surg Res.

[12] Garrone P, Biondi-Zoccai G, Salvetti I, Sina N, Sheiban I, Stella PR, Agostoni P (2009). Quantitative coronary angiography in the current era: principles and applications. J Interv Cardiol.

[13] Milzi A, Dettori R, Marx N, Reith S, Burgmaier M (2021). Quantitative flow ratio (QFR) identifies functional relevance of non-culprit lesions in coronary angiographies of patients with acute myocardial infarction. Clin Res Cardiol.

[14] Suzuki N, Asano T, Nakazawa G (2020). Clinical expert consensus document on quantitative coronary angiography from the Japanese Association of Cardiovascular Intervention and Therapeutics. Cardiovasc Interv Ther.

[15] Milzi A, Dettori R, Burgmaier K, Marx N, Reith S, Burgmaier M (2021). Quantitative flow ratio is related to intraluminal coronary stenosis parameters as assessed with optical coherence tomography. J Clin Med.

[16] Abbasi SH, Sundin Ö, Jalali A, Soares J, Macassa G (2018). Ethnic differences in the risk factors and severity of coronary artery disease: a patient-based study in Iran. J Racial Ethn Health Disparities.

[17] Lee J, Seo KW, Yang HM (2020). Comparison of three-dimensional quantitative coronary angiography and intravascular ultrasound for detecting functionally significant coronary lesions. Cardiovasc Diagn Ther.

[18] Neleman T, van Zandvoort LJ, Tovar Forero MN (2022). FFR-guided PCI optimization directed by high-definition IVUS versus standard of care: the FFR REACT trial. JACC Cardiovasc Interv.

[19] Mentias A, Sarrazin MV, Saad M, Panaich S, Kapadia S, Horwitz PA, Girotra S (2020). Long-term outcomes of coronary stenting with and without use of intravascular ultrasound. JACC Cardiovasc Interv.

[20] Koo BK, Hu X, Kang J (2022). Fractional flow reserve or intravascular ultrasonography to guide PCI. N Engl J Med.

[21] Yang HM, Lim HS, Seo KW (2018). Intravascular ultrasound characteristics in patients with intermediate coronary lesions and borderline fractional flow reserve measurements. Medicine (Baltimore).

[22] Collet C, Onuma Y, Sonck J (2018). Diagnostic performance of angiography-derived fractional flow reserve: a systematic review and Bayesian meta-analysis. Eur Heart J.

[23] Song L, Xu B, Tu S (2022). 2-year outcomes of angiographic quantitative flow ratio-guided coronary interventions. J Am Coll Cardiol.

[24] Tanigaki T, Emori H, Kawase Y (2019). QFR versus FFR derived from computed tomography for functional assessment of coronary artery stenosis. JACC Cardiovasc Interv.

[25] Peper J, van Hamersvelt RW, Rensing BJ (2021). Diagnostic performance and clinical implications for enhancing a hybrid quantitative flow ratio-FFR revascularization decision-making strategy. Sci Rep.

